# P35 Lancashire and South Cumbria BLING Project

**DOI:** 10.1093/jacamr/dlag102.041

**Published:** 2026-06-26

**Authors:** Shaun Morgan, Kelly Newby, Kerri Robinson, Chris Shaw, Hisham Ziglam

**Affiliations:** Lancashire and South Cumbria Critical Care Network, Preston, UK; University Hospitals of Morecambe Bay NHS Foundation Trust, Barrow-in-Furness, UK; East Lancashire Hospitals NHS Trust, Blackburn, UK; Department of Critical Care, East Lancashire Hospitals NHS Trust, Blackburn, UK; East Lancashire Hospitals NHS Trust, Blackburn, UK

## Abstract

**Background:**

Continuous and extended β-lactam infusions offer pharmacokinetic advantages for critically unwell adults but require operational change. Following publication of the BLING III trial and the 2025 BIA/ICS position statement, the Lancashire and South Cumbria Critical Care Network sought to standardize β-lactam administration across the region to reduce variation, improve safety, and integrate sustainability considerations.

**Objectives:**

(i) Develop and implement a unified regional guideline for meropenem and piperacillin/tazobactam administration in adult critical care aligned with BIA/ICS recommendations. (ii) Assess and minimize the environmental impact of β-lactam administration through optimization of diluent and plastic use. (iii) Deliver coordinated education and training to the multidisciplinary workforce, including 414 critical care nurses, to support consistent implementation.

**Methods:**

A multidisciplinary working group (Antimicrobial Stewardship, pharmacy, critical care) oversaw the project. Stakeholder engagement was achieved through educational sessions on BLING III. A benchmarking exercise across five critical care units assessed existing administration practices. Sustainability modelling compared carbon lifecycle impact between infusion strategies. Guideline drafts underwent review through local and regional governance committees to ensure safety, feasibility, and operational clarity. Educational resources and training sessions were developed to support implementation.

**Results:**

Three differing administration strategies for β-lactams were identified regionally, with variation in diluents, infusion volumes, and delivery methods. The network agreed to adopt continuous and extended infusion strategies prior to national guidance publication, anticipating BLING III and BIA/ICS recommendations. A unified guideline was co-developed over several months, incorporating creatinine-clearance–adjusted dosing and recommendations for renal replacement therapy. Sustainability analyses predicted an approximate 37.9% CO₂e reduction per piperacillin/tazobactam course with the new administration strategy due to reduced plastic and diluent use. The guideline launched during World AMR Awareness Week 2025, supported by a structured education package, standardized teaching materials, and region-wide training. Early feedback from clinical teams highlighted improved clarity, consistency, and confidence in β-lactam administration.

**Conclusions:**

This collaborative regional project demonstrates a scalable approach to implementing continuous and extended β-lactam infusions within adult critical care. Standardization reduced practice variability, supported safe and evidence-based dosing, and incorporated sustainability gains alongside workforce education. The initiative provides an exemplar for antimicrobial stewardship teams seeking to embed impactful, system-level practice change that enhances antimicrobial optimization and patient outcomes.

